# Cecropin A: investigation of a host defense peptide with multifaceted immunomodulatory activity in a chicken hepatic cell culture

**DOI:** 10.3389/fvets.2024.1337677

**Published:** 2024-03-01

**Authors:** Rege Anna Márton, Csilla Sebők, Máté Mackei, Patrik Tráj, Júlia Vörösházi, Ágnes Kemény, Zsuzsanna Neogrády, Gábor Mátis

**Affiliations:** ^1^Division of Biochemistry, Department of Physiology and Biochemistry, University of Veterinary Medicine, Budapest, Hungary; ^2^National Laboratory of Infectious Animal Diseases, Antimicrobial Resistance, Veterinary Public Health and Food Chain Safety, University of Veterinary Medicine, Budapest, Hungary; ^3^Department of Pharmacology and Pharmacotherapy, Medical School, University of Pécs, Pécs, Hungary; ^4^Department of Medical Biology, Medical School, University of Pécs, Pécs, Hungary

**Keywords:** antimicrobial peptide, host defense peptide, cecropin A, poultry, hepatic cell culture, immunomodulatory

## Abstract

**Introduction:**

Host defense peptides (HDPs) are increasingly referred to as promising candidates for the reduction of the use of conventional antibiotics, thereby combating antibiotic resistance. As HDPs have been described to exert various immunomodulatory effects, cecropin A (CecA) appears to be a potent agent to influence the host inflammatory response.

**Methods:**

In the present study, a chicken primary hepatocyte–non-parenchymal cell co-culture was used to investigate the putative immunomodulatory effects of CecA alone and in inflammatory conditions evoked by polyinosinic-polycytidylic acid (Poly I:C). To examine the viability of the cells, the extracellular lactate dehydrogenase (LDH) activity was determined by colorimetric assay. Inflammatory markers interleukin (IL)-8 and transforming growth factor-ß1 (TGF-ß1) were investigated using the ELISA method, whereas concentrations of IL-6, IL-10, and interferon-γ (IFN-γ) were assayed by Luminex xMAP technology. Extracellular H_2_O_2_ and malondialdehyde levels were measured by fluorometric and colorimetric methods, respectively.

**Results:**

Results of the lower concentrations suggested the safe application of CecA; however, it might contribute to hepatic cell membrane damage at its higher concentrations. We also found that the peptide alleviated the inflammatory response, reflected by the decreased production of the pro-inflammatory IL-6, IL-8, and IFN-γ. In addition, CecA diminished the levels of anti-inflammatory IL-10 and TGF-ß1. The oxidative markers measured remained unchanged in most cases of CecA exposure.

**Discussion:**

CecA displayed a multifaceted immunomodulatory but not purely anti-inflammatory activity on the hepatic cells, and might be suggested to maintain the hepatic inflammatory homeostasis in Poly I:C-triggered immune response. To conclude, our study suggests that CecA might be a promising molecule for the development of new immunomodulatory antibiotic-substitutive agents in poultry medicine; however, there is still a lot to clarify regarding its cellular effects.

## 1 Introduction

The indiscriminate use of conventional antibiotics, both in human and veterinary medicine, has contributed to the global spread of antibiotic resistance, and based on the predictions of the World Health Organization (WHO), it could claim over 10 million human lives by 2050 (1). Therefore, there is an urgent demand to search for alternatives that can provide a novel antimicrobial mode of action (1, 2). Finding a potential replacement is crucial for livestock farming also, where animals are largely exposed to pathogens, while production efficiency, as well as animal health and well-being, have to be maintained (2). In addition, antibiotic resistance can also pose a serious risk to handlers of farm animals and consumers of animal products, further contributing to the need for the development of novel antimicrobial substances (3). In this field, host defense peptides (HDPs) – originally known as antimicrobial peptides (AMPs) – have recently aroused great interest and seem to be promising candidates for designing new antimicrobial agents (1).

HDPs are generally small, cationic peptides consisting of 10–50 amino acids and produced by every living organism as an essential part of their innate immune system, thereby helping the host to overcome infections originating from various pathogens (1, 4). Initial studies aimed to discover the common structural and physiological characteristics necessary for their direct microbicidal activity (5). Based on these findings, it is now well-documented that HDPs display broad-spectral antimicrobial effects, even against multi-resistant bacteria, viruses, fungi, or protozoa (4). However, besides owning the ability to directly attack microbes, it has recently attracted more attention that HDPs are able to influence the host immune response, thereby offering alternative mechanisms to combat infections (1, 5). This can be achieved in a variety of ways, such as regulating the production of various cytokines, stimulating chemotaxis, supporting immune cell differentiation, promoting wound-healing, exerting an anti-endotoxin effect, inhibiting toll-like receptors (TLR), or shaping the normal microbiota of the gut (6–8). This indirect impact and the capability of acting on multiple targets can be considered one of their greatest advantages over conventional antibiotics (1), and it can be suggested that rather than acting directly, their primary function is to serve as important signaling molecules affecting cellular activities (8).

Due to the widespread occurrence, HDPs of insect origin represent one of the largest groups, among which cecropins are extensively studied peptides (1, 2, 9). Apart from having already proven broad-spectral antimicrobial feature, especially against Gram-negative bacteria (9), several cecropins and cecropin-like HDPs have been described to have beneficial effects on the host, such as immunomodulatory (10–13), antioxidant (14–17), or antitumor activity (9), and improvement of the intestinal epithelial integrity and morphology (2, 15, 18). Moreover, some of them can act even as growth promoters in farm animals in various ways (2, 19, 20). It has been reported that certain of these advantageous outcomes also pertain to cecropin A (CecA) (21–24), a 37 amino acid-containing natural insect HDP from the family of cecropins (23). Acting mainly as an anti-inflammatory agent, CecA was able to improve survival after *Escherichia coli*-induced peritonitis in mice, decreasing the endotoxin and tumor necrosis factor α (TNFα) concentrations in the blood (25). Furthermore, it could alleviate inflammation in experimentally induced inflammatory bowel disease (IBD) of mice by hindering the production of pro-inflammatory TNFα, interleukin-1ß (IL-1ß), and IL-6 (21). *In vitro* studies on different cell lines proved that the anti-inflammatory effect of CecA was exhibited by the inhibition of key regulatory proteins of inflammation, such as cyclooxygenase-2 (COX-2) and mitogen-activated protein kinases (MAPKs), which contributed to the reduced production of various pro-inflammatory cytokines (22, 23).

Despite being a thoroughly investigated and promising molecule, only a few studies are available concerning the effects of CecA on a cellular level, and to date, none of them have been carried out on the liver. However, the liver plays a key role in maintaining local and systemic homeostasis by regulating inflammatory processes. The healthy liver is constantly exposed to gut-derived microbial metabolites and components that must be tolerated while also being prepared to react when required. This regular exposure to microbial compounds, paired with a continuously changing microenvironment, results in a strictly controlled immune state (26). Resident immune cells, for instance, Kupffer cells, monocyte-derived macrophages, myeloid cells, or lymphoid cells, are of great importance in the regulation of these inflammatory processes, by detecting pathogen-associated molecular patterns (PAMPs) or damage-associated molecular patterns (DAMPs), interacting with other local cells, or producing inflammatory cytokines and chemokines (26, 27). Apart from them, non-hematopoietic cells, including hepatocytes or hepatic stellate cells (HSC), also have great significance during inflammatory processes, as their surfaces are rich in pattern recognition receptors (PRRs), and they can also produce inflammatory mediators (26, 28).

As the central organ of detoxification and metabolism, the liver is highly vulnerable not only to inflammation but also to oxidative stress caused by free radicals, such as reactive oxygen species (ROS) and reactive nitrogen species (RNS). Apart from this, more and more evidence suggests that inflammation and redox imbalance often play a crucial role in hepatic damage as complex and tightly regulated interacting processes (29). Not only inflammatory response, but also the formation of free radicals is an important mechanism against pathogens. However, their excessive deliberation might have a detrimental impact on liver homeostasis and adverse outcomes for poultry, thus causing immunosuppression, intestinal disorders, and impaired production. Therefore, it is relevant to investigate not only the inflammatory but also the oxidative state (30).

Given the prevalence of antibiotic resistance, the urgent demand to search for novel antimicrobial agents in poultry farming and the versatile immunomodulatory action of HDPs, the investigation of the impact of their representatives at a cellular level is of great importance. As limited data are available about the immunomodulatory role of CecA in domestic animals, and none of them investigated the effects on poultry or hepatic cells, the goal of the present study was to examine the peptide’s influence on the immune response and the redox homeostasis, using a primary hepatocyte–non-parenchymal cell co-culture of chicken origin. Since TLRs, as PRRs, play a crucial role in the recognition of pathogens and induce downstream signaling leading to inflammation (5), the synthetic double-stranded RNA (dsRNA) analog polyinosinic-polycytidylic acid (Poly I:C), as a TLR3-agonist, was used to evoke inflammation, which was previously applied successfully by our research group for this purpose (31–33).

## 2 Materials and methods

### 2.1 Process of cell isolation

A 3 weeks-old male Ross-308 broiler chicken was used for cell isolation. The chicken was kept in the animal house of the Department of Physiology and Biochemistry, University of Veterinary Medicine Budapest, Hungary. The animal was fed according to the breeder’s instructions and water was provided *ad libitum*. All efforts were committed to maintain the circumstances for animal welfare, and our experiments were in line with the laws of the European Union, approved by the Local Animal Welfare Committee, and enabled by the Government Office (number of permission: GK-419/2020; date of approval: 11 May 2020). If not stated otherwise, all the described chemicals and compounds were purchased from Merck KGaA (Darmstadt, Germany).

The process of cell isolation was performed according to Mackei et al., (34). Extermination of the animal was carried out by decapitation, using carbon dioxide narcosis. After the removal of the abdominal feathers and disinfection of the skin, the body cavity was opened, and the portal system was cannulated through the *gastropancreaticoduodenal* vein, using a 22-size venous cannula. Next, the liver was perfused using a three-step perfusion system at a flow rate of 30 mL/min. All solutions were preheated to 40°C and oxygenated by Carbogen (composition: 95% O_2_, 5% CO_2_; flow rate 1 L/min) immediately before use. To begin with, the perfusion was performed by using 150 mL of Hanks’ Balanced Salt Solution (HBSS) buffer, containing ethylene glycol-bis(2-aminoethyl ether)-N,N,N′,N′-tetraacetic acid (EGTA), followed by the application of 150 mL EGTA-free HBSS buffer. As a last step of perfusion, the liver was flushed with 100 mL HBSS solution supplemented with 100 mg of type IV collagenase, 7 mM CaCl_2,_ and 7 mM MgCl_2_. After the removal of the organ and the Glisson’s capsule, the cells were suspended in 50 mL of HBSS buffer supplemented with bovine serum albumin (BSA, 2.5%) to ensure the avoidance of the cells’ aggregation. The resulting cell suspension was filtered through a three-layer sterile gauze sheet and then allowed to stand on ice for 45 min. In the next step, the cell suspension was centrifuged (3 min, 100 × g) three times, during which the resulting supernatant was collected separately, and the sediment was resuspended in Williams’ Medium E (supplemented with 0.22% NaHCO_3_, 50 mg/mL gentamycin, 2 mM glutamine, 4 g/L dexamethasone, 20 IU/L insulin, 5% fetal bovine serum [FBS] and 0.5 g/mL amphotericin B). After centrifugation for the third time, the sediment was resuspended again to obtain a hepatocyte-rich cell suspension. On the other hand, the non-parenchymal cells-containing previously collected supernatants were mixed and centrifuged (10 min, 350 × g). Eventually, the remaining supernatant was centrifuged (10 min, 800 × g), and the pellet was resuspended in Williams Medium E, thereby gaining the non-parenchymal cell-rich fraction. Hepatocytes and macrophages in the former and latter suspensions were previously characterized by immunofluorescent staining and flow cytometry by Mackei et al., (34). In this earlier experiment of our research group, the isolated and cultured hepatocytes were labeled using chicken-specific, fluorescein isothiocyanate (FITC)-coupled anti-albumin, whereas macrophages in the non-parenchymal cell-rich fraction were detected by chicken macrophage-specific phycoerythrin (PE)-conjugated antibodies. The standardized process of cell isolation was performed the same way in the present study, ensuring that the same types of cells were present in the corresponding fractions. After obtaining the cell suspensions, Giemsa staining was performed to check the morphology of the isolated cells and that of confluent cell cultures, confirming their characteristics.

To assess the viability of the cells in both fractions, a trypan blue exclusion test was performed in Bürker’s chambers prior to seeding. Before seeding, the two fractions were diluted according to the cell count, and the hepatocyte-containing suspension was blended with the non-parenchymal cell-rich fraction in a 6 to 1 ratio, receiving a total concentration of 10^6^ cells/mL. With a volume of 400 μL of cell suspension/well, cells were seeded into 24-well culturing plates (Greiner Bio-One Hungary Kft., Mosonmagyaróvár, Hungary) previously coated with rat tail collagen type I. Coating of the plates began with the complete dissolving of 10 mg collagen in 100 mL of 0.1% acetic acid solution. Thereafter, 24-well culturing plates were coated with 300 μL/well (referring to 10 μg/cm^2^), followed by overnight incubation at room temperature and UV light exposure to avoid contamination until complete drying. The first change of the cell culture media was committed after 4 h of incubation at 37°C and 5% CO_2_, which was followed by the treatments after 24 h of incubation under the same conditions.

### 2.2 Treatments

Treatment of the cells was accomplished by using the previously described supplemented cell culture media but without the use of FBS, according to Table 1. The control was created by adding only cell culture media to the cells. To evoke inflammation, Poly I:C was added at a concentration of 50 μg/mL. The effects of CecA were examined solely and in Poly I:C-induced inflammation, respectively, at five different concentrations being 1, 3.125, 6.25, 12.5, and 25 μg/mL. When administering combinatory treatments of Poly I:C and CecA, the two substances were applied at once. Following 24 h treatments, cell culture media samples of 24-well microplates were taken and centrifuged (5 min, 4,000 × g), and aliquots were frozen at −80°C until the below-mentioned measurements.

**Table 1:** Treatment groups applied on primary chicken hepatocyte-non-parenchymal cell co-culture.

Treatment group	Cecropin A	Poly I:C
Control	–	–
Cec-1	1 μg/mL	–
Cec-2	3.125 μg/mL	–
Cec-3	6.25 μg/mL	–
Cec-4	12.5 μg/mL	–
Cec-5	25 μg/mL	–
PI:C	–	50 μg/mL
PI:C + Cec-1	1 μg/mL	50 μg/mL
PI:C + Cec-2	3.125 μg/mL	50 μg/mL
PI:C + Cec-3	6.25 μg/mL	50 μg/mL
PI:C + Cec-4	12.5 μg/mL	50 μg/mL
PI:C + Cec-5	25 μg/mL	50 μg/mL

Cec-1-5, different concentrations of Cecropin A (CecA) supplementation; PI:C, addition of polyinosinic-polycytidylic acid (Poly I:C), 50 μg/mL.

### 2.3 Measurements

#### 2.3.1 Cellular viability

In order to investigate the viability of the cells, the cellular membrane integrity was examined. To achieve this, the Lactate Dehydrogenase Activity Assay Kit (Cat. Nr. MAK066-1KT) was applied to detect lactate dehydrogenase (LDH) released into the culture media from damaged cells. The enzyme reduces NAD^+^ to NADH^+^, which can be specifically detected by colorimetric assay at 450 nm. A 96-well microplate was loaded with 50 μL of culture media samples diluted by LDH Assay Buffer and then supplemented with 50 μL of freshly prepared Master Reaction Mix. The first read of absorbance was performed after 2 min of incubation at 37°C, by using a Multiscan GO 3.2 reader (Thermo Fisher Scientific Inc., Waltham, MA, United States) at 450 nm. Measurements were continued every 5 min until the value of the most active sample was greater than the value of the highest standard.

#### 2.3.2 Inflammatory markers

Based on our results regarding cellular viability, inflammatory and redox markers continued to be examined only in treatment groups containing CecA at concentrations of 1, 3.125, and 6.25 μg/mL, respectively. IL-8 (often referred to as CXCLi2 in chicken) and transforming growth factor-ß1 (TGF-ß1) were measured in the culture media by chicken-specific ELISA kits (MyBioSource Inc., San Diego, CA, United States, Cat. Nr. MBS289628 and MBS261515, respectively), using a sandwich technique for the former and a double antibody sandwich technique for the latter. Steps were carried out according to the manufacturer’s instructions, and absorbance values were read by a Multiscan GO 3.2 reader at 450 nm.

Levels of IL-6, IL-10, and interferon-γ (IFN-γ) were assayed by Luminex xMAP technology, using Milliplex Chicken Cytokine/Chemokine Panel (Merck KGaA Cat. Nr.: GCYT1-16 K). According to the instructions of the manufacturer, a 96-well microplate attached to the kit was loaded with 25 μL of cell culture media sample/well, using duplicates. Thereafter, 25 μL of three colored capture antibody-coated bead sets was added to each well. After overnight incubation and washing, biotinylated detection antibody and streptavidin phycoerythrin were added to the plate. As a next step, 150 mL of drive fluid was added, followed by the resuspension of beads for 5 min on a plate shaker. Reading was performed using Luminex MAGPIX^®^ instrument, and data were collected by Luminex xPonent 4.2 program. According to bead median fluorescence intensity, standard curves were generated by Belysa Immunoassay Curve Fitting software (Merck KGaA, Darmstadt, Germany) for all analytes.

#### 2.3.3 Redox markers

Extracellular (EC) hydrogen peroxide level was measured in the culture media, using the fluorometric Amplex Red method (Thermo Fisher Scientific, Waltham, MA, United States, Cat. Nr. A21188), according to the protocol provided by the manufacturer. A 96-well microplate was loaded with 50 μL of culture media samples, followed by the addition of 50 μL of prior-to-use-prepared Amplex Red Working solution. After 30 min long incubation at room temperature, fluorescence values were obtained by a Victor X2 2030 fluorometer (Perkin Elmer Inc., Waltham, MA, United States), at wavelengths of 530 nm (excitation) and 590 nm (emission).

In order to determine the extent of lipid peroxidation, the malondialdehyde (MDA) concentration of the cell culture media was measured with Lipid Peroxidation (MDA) Assay Kit (Cat. Nr. MAK085-1KT). According to the protocol attached by the manufacturer, 150 μL of thiobarbituric acid (TBA) solution and 50 μL of each sample were mixed and incubated for 1 h at 95°C, thereby allowing the formation of an MDA-TBA complex. After cooling down to room temperature, a 96-well microplate was loaded with 200 μL of the mixture. Absorbance values were measured at 532 nm, by using a Multiscan GO 3.2 reader.

### 2.4 Statistical analysis

Statistical analysis of data was carried out by using R v. 4.0.3 (R Core Team, 2020). Wilcoxon signed-rank test was performed for pairwise comparisons, given that Shapiro–Wilk tests indicated that the data of several treatment groups had non-normal distributions. If the calculated *p*-value was lower than 0.05, the difference was considered significant. The treatment groups containing different concentrations of solely applied CecA and the inflammatory control where Poly I: C was used alone were compared to the control group. When CecA was administered together with Poly I:C, the results were collated with the Control and also with the group treated only with Poly I:C. Graphs were created using Prism 9 (GraphPad Software Inc., San Diego, CA, V 9.2.1).

## 3 Results

### 3.1 Cellular viability

**Figure 1:**
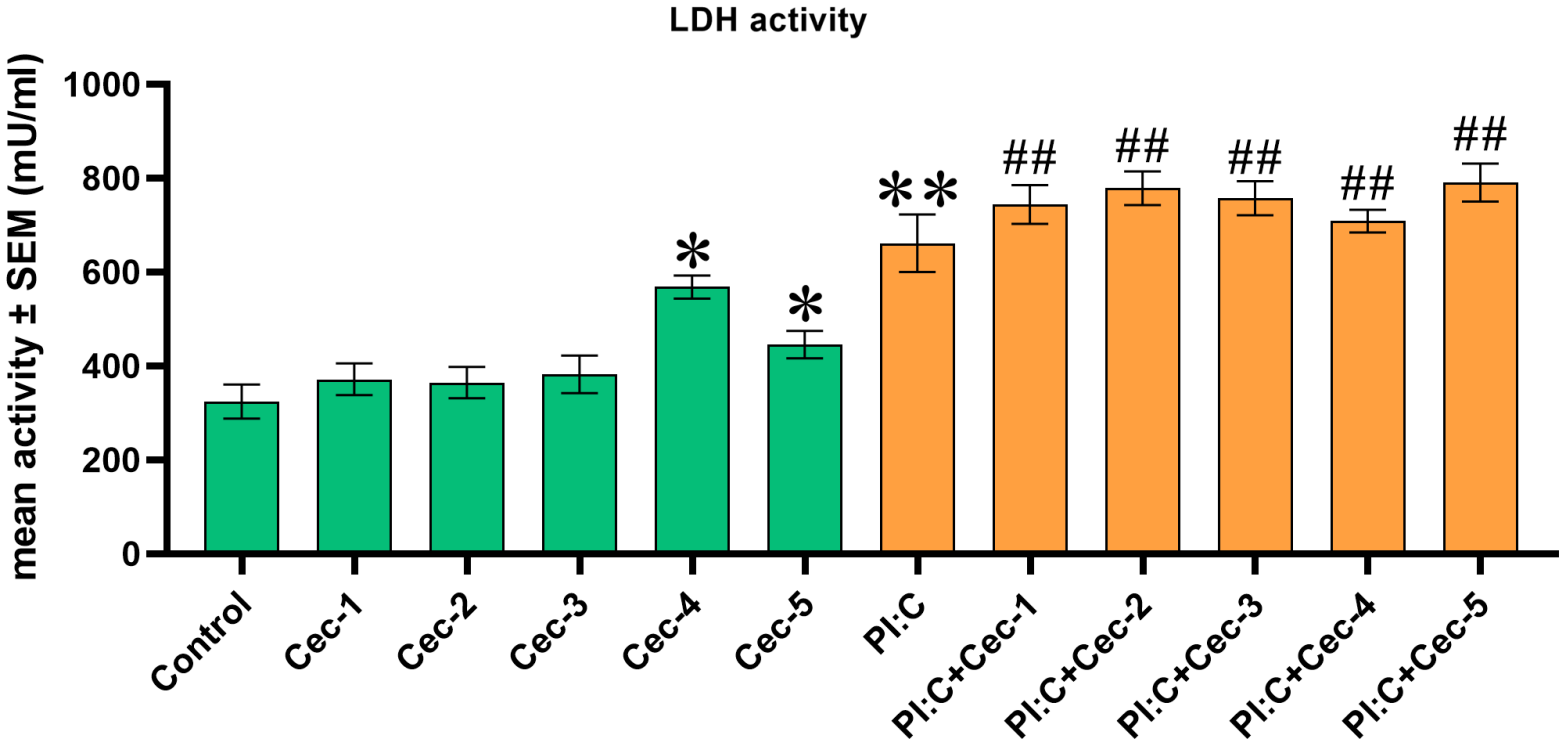
Bar graph showing extracellular lactate dehydrogenase (LDH) activity measured by colorimetric assay. Chicken hepatocyte-non-parenchymal cell co-cultures were treated with five different concentrations of cecropin A (CecA) alone and in combination with polyinosinic-polycytidylic acid (Poly I:C). Green color refers to treatment groups without the addition of Poly I:C, while orange color refers to treatment with Poly I:C. Columns represent means ± SEM (*n* = 6/treatment group). Cec-1 = 1 μg/mL CecA, Cec-2 = 3.125 μg/mL CecA, Cec-3 = 6.25 μg/mL CecA, Cec-4 = 12.5 μg/mL CecA, Cec-5 = 25 μg/mL CecA, PI:C = 50 μg/mL Poly I:C. Cells receiving none of the treatments are considered as Control. Asterisks indicate significant differences when treatment groups Cec-1, Cec-2, Cec-3, Cec-4, Cec-5, and PI:C were compared to Control, whereas combinations of Poly I:C and CecA (PI:C + Cec-1, PI:C + Cec-2, PI:C + Cec-3, PI:C + Cec-4, PI:C + Cec-5) were compared to the group PI:C. Hashtags indicate significant differences when comparing combinations of Poly I:C and CecA to Control. *p < 0.05, **p < 0.01, and ^##^*p* < 0.01.

To assess cell membrane damage, EC LDH activity was determined. Cells treated only with CecA showed a significant increase of enzyme activity at the two highest concentrations (12.5 and 25 μg/mL) of the peptide (*p* = 0.0159 and *p* = 0.0381, respectively), whereas the 1, 3.125, and 6.25 μg/mL concentrations did not seem to affect the cell membrane integrity. Poly I:C exerted a significant increase in LDH activity compared to the control group (*p* = 0.0095), which was affected by neither of the applied concentrations of CecA. However, significantly higher values were observed when combining Poly I:C and each concentration of the peptide (*p* = 0.0095 for all five comparisons), compared to the control group (Figure 1).

### 3.2 Inflammatory markers

**Figure 2:**
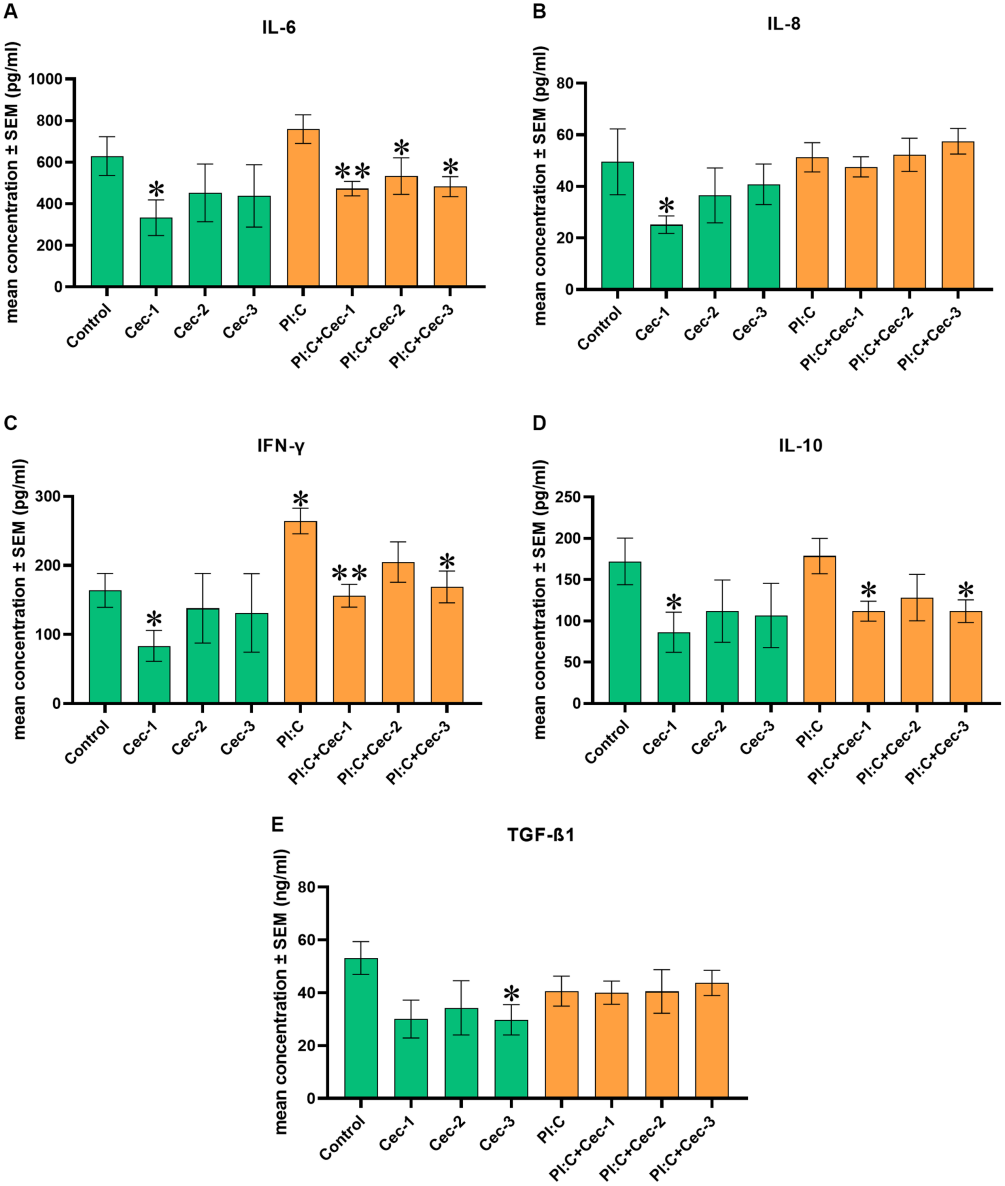
Bar graphs showing **(A)** IL-6 concentration measured by chicken-specific Luminex MAGPIX Panel. **(B)** IL-8 concentration measured by chicken-specific ELISA. **(C)** IFN-γ concentration measured by chicken-specific Luminex MAGPIX Panel. **(D)** IL-10 concentration measured by chicken-specific Luminex MAGPIX Panel. **(E)** TGF-ß1 concentration measured by chicken-specific ELISA. Chicken hepatocyte-non-parenchymal cell co-cultures were treated with three different concentrations of cecropin A (CecA) alone and in combination with polyinosinic-polycytidylic acid (Poly I:C). Green color refers to treatment groups without the addition of Poly I:C, while orange color refers to treatment with Poly I:C. Columns represent means ± SEM (*n* = 6/treatment group). Cec-1 = 1 μg/mL CecA, Cec-2 = 3.125 μg/mL CecA, Cec-3 = 6.25 μg/mL CecA, PI:C = 50 μg/mL Poly I:C. Cells receiving none of the treatments are considered as Control. Asterisks indicate significant differences between treatment groups. Groups Cec-1, Cec-2, Cec-3 and PI:C were compared to Control, whereas combinations of Poly I:C and CecA (PI:C + Cec-1, PI:C + Cec-2, PI:C + Cec-3) were compared to the group PI:C. *p < 0.05 and **p < 0.01.

In order to investigate the impact of CecA on the immune response, the levels of IL-6, IL-8, IFN-γ, IL-10, and TGF-ß1 were determined. When measuring IL-6, CecA alone at 1 μg/mL was able to decrease the level of the cytokine (*p* = 0.0381). Compared to the inflammation evoked by Poly I:C, concentrations of 1, 3.125, and 6.25 μg/mL of CecA attenuated the production of IL-6 (*p* = 0.0022, *p* = 0.0411, and *p* = 0.0152, respectively) (Figure 2A).

In the case of IL-8, solely applied CecA in 1 μg/mL contributed to a significant decrease (*p* = 0.0381), whereas at concentrations of 3.125 and 6.25 μg/mL, no significant changes were observed. Neither Poly I:C alone nor the combined treatments of Poly I:C and the different concentrations of CecA affected the amount of IL-8 (Figure 2B).

Regarding IFN-γ, the sole administration of CecA at 1 μg/mL diminished the level of the cytokine (*p* = 0.0381). On the contrary, Poly I: C significantly elevated the level of IFN-γ (*p* = 0.0191), which was attenuated by CecA at 1 and 6.25 μg/mL (*p* = 0.0022 and *p* = 0.0152, respectively) (Figure 2C).

The level of IL-10 was found to be decreased only by the lowest dose (1 μg/mL) of solely applied CecA (*p* = 0.0381). Combinations of Poly I:C and the 1 μg/mL, as well as the 6.25 μg/mL concentrations of CecA also contributed to a significant reduction of the amount of the cytokine (*p* = 0.0260 and *p* = 0.0411, respectively) (Figure 2D).

In addition, when measuring TGF-ß1, solely applied CecA in 6.25 μg/mL contributed to a significant decrease in its level (*p* = 0.0416), whereas at concentrations of 1 and 3.125 μg/mL, no significant changes were observed. Neither Poly I:C alone nor the combined treatments of Poly I:C and the different concentrations of CecA affected the amount of TGF-ß1 (Figure 2E).

### 3.3 Redox markers

**Figure 3:**
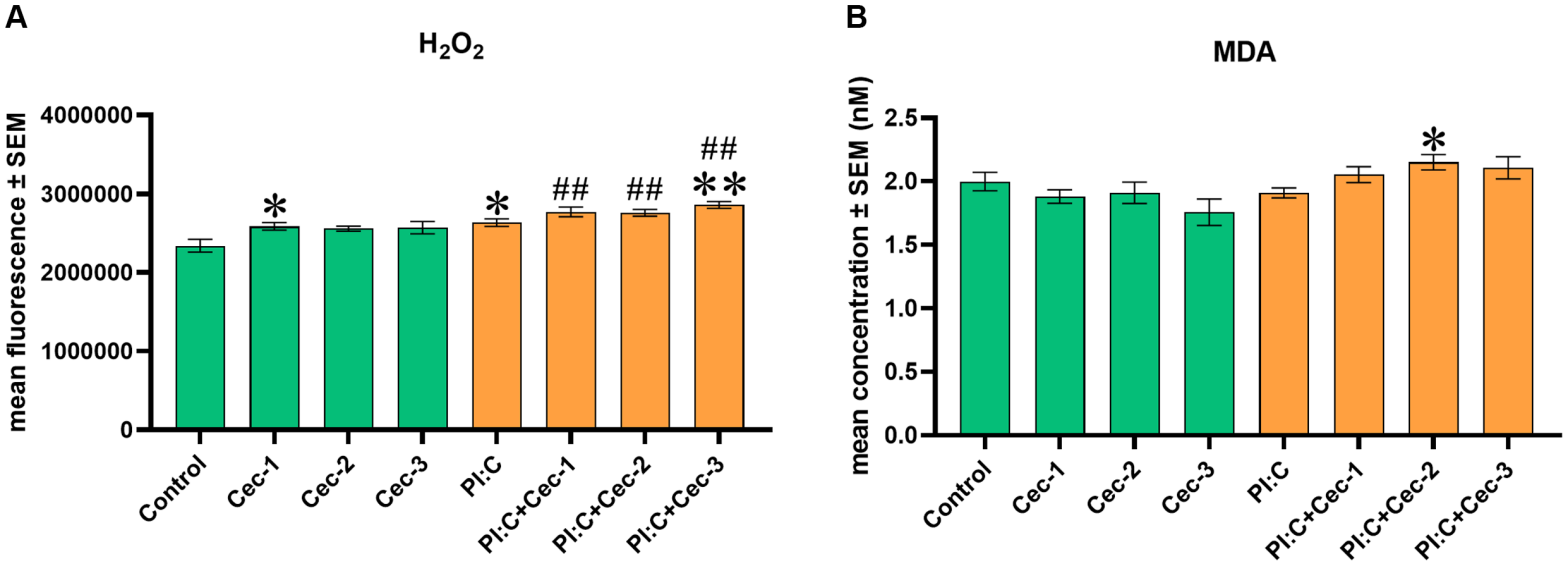
Bar graph showing **(A)** EC H_2_O_2_ concentration measured by fluorometric assay. **(B)** MDA concentration measured by colorimetric assay. Chicken hepatocyte-non-parenchymal cell co-cultures were treated with three different concentrations of cecropin A (CecA) alone and in combination with polyinosinic-polycytidylic acid (Poly I:C). Green color refers to treatment groups without the addition of Poly I:C, while orange color refers to treatment with Poly I:C. Columns represent means ± SEM (*n* = 6/treatment group). Cec-1 = 1 μg/mL CecA, Cec-2 = 3.125 μg/mL CecA, Cec-3 = 6.25 μg/mL CecA, PI:C = 50 μg/mL Poly I:C. Cells receiving none of the treatments are considered as Control. Asterisks indicate significant differences when treatment groups Cec-1, Cec-2, Cec-3 and PI:C were compared to Control, whereas combinations of Poly I:C and CecA (PI:C + Cec-1, PI:C + Cec-2, PI:C + Cec-3) were compared to the group PI:C. Hashtags indicate significant differences when comparing combinations of Poly I:C and CecA to Control. *p < 0.05, **p < 0.01, and ^##^*p* < 0.01.

For the examination of the effect of CecA on redox homeostasis, the level of EC H_2_O_2_ and the MDA concentration indicating lipid peroxidation were measured. In the case of the H_2_O_2_ level, the lowest administered dose of CecA (1 μg/mL) was observed to enhance the amount of the oxidative marker (*p* = 0.0381), whereas the other applied concentrations of the peptide did not seem to affect it. When evoking inflammation, Poly I:C alone significantly increased the level of H_2_O_2_ (*p* = 0.0381), which elevation was further enhanced by CecA at a concentration of 6.25 μg/mL (*p* = 0.0087); however, no significant changes were observed concerning the treatment groups with concentrations of 1 or 3.125 μg/mL of the HDP. In addition, significantly higher values were observed when combining Poly I:C and each concentration of the peptide (*p* = 0.0095 for all three comparisons), compared to the control group (Figure 3A).

Regarding MDA, neither of the solely applied concentrations of CecA influenced the marker of lipid peroxidation. On the other hand, in Poly I:C-induced inflammation, CecA contributed to a significant elevation at its concentration of 3.125 μg/mL (*p* = 0.0123) (Figure 3B).

## 4 Discussion

As the emergence of antibiotic resistance has become a global concern, there is an outstanding need for the design of new antimicrobial agents (5). For this purpose, HDPs have attracted great attention, even from poultry farming (1), where animals are constantly challenged by a large set of pathogens. A growing number of studies have shown that, in addition to their direct antimicrobial effect, HDPs possess remarkable immunomodulatory properties (5, 7, 8). However, rather than acting on a single receptor or signaling pathway, HDPs exert a pleiotropic effect and selectively influence certain immune processes (7). As a consequence, the investigation of their cellular effects is crucial for their therapeutic use in the future, thereby contributing to the fight against antibiotic resistance.

In the present study, the immunomodulatory effects of CecA were examined solely and in inflammatory conditions induced by Poly I:C, on a primary hepatocyte–non-parenchymal cell co-culture of chicken origin. Being a synthetic dsRNA molecule, Poly I:C acts as a potent agonist of TLR3 (33), the immunomodulatory role of which has already been confirmed also in chickens (35). Upon stimulation of the receptor, downstream signaling promotes the activation of nuclear factor-κB (NF-κB) signal transduction and further intracellular signals, leading to the production of proinflammatory cytokines (for example type I interferons) and the maturation of dendritic cells, thereby contributing to inflammation (33). As recent evidence suggests that similar to mammalians, NF-κB signaling plays an important role in the inflammatory state of poultry as well (30), Poly I:C might be an adequate candidate to evoke inflammation in chicken cell cultures. In the present study, Poly I:C contributed to increased production of IFN-γ, indicating the triggered inflammatory state.

In order to confirm the possible safe use of HDPs in the future, their potential cytotoxic effects on the host cells must be thoroughly investigated (36). Still, limited data are available concerning their interactions with the eukaryotic cells (24). As cationic HDPs are usually described as having high selectivity to bacteria (36), it has also been highlighted that cecropins possess low cytotoxicity against mammalian cells (9). Even so, these findings have not been confirmed in the case of poultry. According to our results, the treatment of cells with CecA at lower concentrations did not result in a change in cell membrane integrity, suggesting its safe application. This is in line with former findings, as CecA was not found to have unwanted effects against murine macrophage cell line RAW 264.7 (23), porcine intestinal epithelial cell line IPEC-J2 (22), and human peripheral mononuclear cell cultures (36). However, in our study, the higher concentrations of the peptide caused a significant increase in the EC LDH activity, indicating cell membrane leakage and a decrease in cell viability. Even though there are data available where a higher administered dose of CecA or its synthetic derivative displayed cytotoxic effects (24, 37–39), the role of membrane damage in it is controversial. Nevertheless, CecA’s contribution to cell death was described to befall via a caspase-independent apoptotic effect rather than causing necrosis (24, 36). Furthermore, according to literature data, even the same concentrations of the peptide displayed diverse effects on the viability of different cell types. Hence, the cytotoxicity of CecA might also depend on the examined cell type and the origin of the used cell culture. In addition, low tissue penetration of HDPs after their addition might result in low availability in the organs (40), thus, achieving a cytotoxic dose under *in vivo* conditions may be less likely to occur in the liver. Taking our data together, the administration of CecA still appears to be harmless to the host cells at low concentrations; however, from a hepatic perspective, avoiding higher concentrations of it might be advisable to consider.

Immunomodulatory effects of CecA were investigated by the measurement of different cytokines such as IL-6, IL-8, IFN-γ, TGF-ß1, and IL-10. While IL-6, IL-8, and IFN-γ are usually described as pro-inflammatory mediators, produced by a large set of cells (41), IL-10 and TGF-ß are mainly considered anti-inflammatory (28, 41, 42). IL-6 plays a crucial role in inflammation, as it is involved in the recruitment of leukocytes, acute-phase protein production of hepatocytes, proliferation of T cells, and differentiation of B cells (41). IL-8 also has an important role in the recruitment of neutrophils, natural killer (NK) cells, and T cells to the site of injury (41). Furthermore, IFN-γ is a key regulator of macrophage activation, antigen presentation, and cytotoxic cellular responses (41).

In the present study, the single dose of CecA at 1 μg/mL and the administration of the HDP in Poly I:C-induced inflammation at all three concentrations resulted in a decrease of IL-6. This is in accordance with previous findings, where CecA was able to inhibit the production of the cytokine in IPEC-J2 cell line co-cultured with *Escherichia coli*, thereby alleviating inflammation (22). Other natural cecropins such as SibaCec (12), *Musca domestica* cecropin (Mdc) (15), cecropin-TY1 (10), and *Aedes egypti* cecropins (11) were also found to exert the same effect in different cell cultures. Moreover, under *in vivo* conditions, in experimentally induced IBD of mice, CecA has been shown to relieve symptoms and reduce the local level of IL-6 (21). In the case of IFN-γ, a significant reduction was measured after the sole addition of CecA at 1 μg/mL and after Poly I:C-evoked inflammation at concentrations of 1 and 6.25 μg/mL, respectively. Although to the best of the authors’ knowledge, the effect of CecA on IFN-γ production has not been previously investigated, our findings are in line with the results of other cecropins. Namely, Mdc in an experimental mice model (15) and cecropin AD in turbot fish (*Scophthalmus maximus*) (18) were also able to alleviate the level of IFN-γ under *in vivo* conditions. Regarding IL-8, CecA displayed a reducing effect at the sole concentration of 1 μg/mL in our study, which is in accordance with the findings of Zhai et al., where a similar impact was observed in IPEC-J2 cell line co-cultured with *Escherichia coli* (22). Taking the above-mentioned data together, our results suggest the anti-inflammatory activity of CecA, the mechanism of which has already been proven to be achieved by the inhibition of such key proteins of inflammation as COX-2 and MAPKs (23).

IL-10, an anti-inflammatory cytokine, is produced in response to microbial stimuli mainly by macrophages and dendritic cells, and exerts a direct suppressive effect primarily on their cellular level (42). It serves as a key regulator of the inflammatory balance, as the immune response must protect the organism without contributing to excessive reaction and immunopathology (42). In our experiment, the levels of IL-10 were diminished by the solely applied CecA at 1 μg/mL and the treatment of the peptide in Poly I:C-induced inflammation at 1 and 6.25 μg/mL. Although to the best of the authors’ knowledge, the effect of CecA on IL-10 production has not been previously investigated, our findings are in line with the results of Mdc, another cecropin in an experimentally induced IBD of mice (15). As IL-10 production demands, among others, the activation of p38 MAPK and extracellular signal-regulated kinase (ERK) (42), and CecA exerts an inhibitory effect on these key proteins (23), the decrease in the IL-10 level might be achieved due to this mechanism. It could also indicate that the immune cells did not require significant amounts of anti-inflammatory cytokines to alleviate the immune response, since CecA had notably decreased the levels of pro-inflammatory ones (43).

TGF-ß is a less frequently examined cytokine in connection with cecropins; however, it possesses multifaceted impacts on the cells, often described as a pleiotropic molecule (44, 45). Besides having a mainly anti-inflammatory effect, which has already been investigated in broiler chickens (46), TGF-ß plays an important role in the regulation of cell proliferation and differentiation, cytokine production, fibrotic processes, repair mechanisms, and forming of the extracellular matrix (28, 44, 45). In addition, it can be produced by various cell types in the liver, with the main sources being HSCs, macrophages, and lymphocytes (28). According to our results, the release of TGF-ß1, an isoform of TGF-ß, can be decreased by the single addition of CecA at 6.25 μg/mL, but not affected by the addition of the peptide in an inflammatory state. To the best of the authors’ knowledge, the effect of CecA on TGF-ß production has not been previously investigated; however, Han and Sheperd made the same observation recently, treating a rainbow trout epithelial-like cell line (RTgill-W1) with another natural cecropin, cecropin P1 (47). Nonetheless, it has been previously published that TGF-ß might have special roles from a hepatic point of view, as it was found that murine and human hepatocytes might be especially responsive to the cytokine, and a high amount of TGF-ß could display an apoptotic effect (28, 48). Therefore, its inhibition might result in the preservation and protection of the hepatic function, moreover, it could also support the apoptosis of HSCs with pathologic phenotype (49). According to our results, by reducing the amount of TGF-ß1 in healthy cells, CecA might exert the same beneficial effects, which might be achieved by the previously mentioned ability to inhibit MAPKs (22, 23), as TGF-ß production is also regulated by this signaling pathway (44).

Taking our results regarding inflammatory markers together, CecA displayed on one hand, an anti-inflammatory effect, as it contributed to decreased production of the pro-inflammatory mediators IL-6, IL-8, and IFN-γ. On the other hand, it also caused a reduction in the levels of the anti-inflammatory IL-10 and TGF-ß1, based on which a purely anti-inflammatory effect of CecA cannot be stated in our experimental circumstances. Even though it is contrary to previous findings, where CecA was clearly characterized as an anti-inflammatory molecule (21–23), it is also worth noting that HDPs are described as immunomodulatory molecules with pleiotropic effects, allowing them to exert even pro-inflammatory activity (7). For instance, the same simultaneous reduction of IL-6 and IL-10 was observed by Hansen et al. when examining the immunomodulatory effects of HDP GKY25 on a RAW 264.7 cell line (50). As a consequence, their effect is not universal and cannot be predicted for a given HDP (5), as it is highly complex and depends on the specific biological context, cell type, and inflammatory stimulus (5, 8). In addition, while the majority of cecropins are described in the literature as possessing anti-inflammatory activity, one can also find example among them for exerting even pro-inflammatory effects, like in the case of cecropin P1 (47). Besides, the present study is subject to limitations, such as the relatively narrow range of inflammatory parameters tested, and the use of only one type of TLR-agonist to induce inflammation.

As more and more evidence suggests that inflammation and redox state are tightly connected to each other (29), and several cecropins have been described to influence the oxidative conditions of the host cells (14–17, 51, 52), the effects of CecA on redox homeostasis were also examined in the present study. According to our results, the low dose of solely applied CecA enhanced the level of EC H_2_O_2,_ and in inflammatory conditions, its high dose also contributed to oxidative stress. Since the excessive formation of ROS contributes to lipid peroxidation (29), levels of MDA were also measured. Neither of the solely added concentrations of CecA was found to increase its amount, which indicates that even though an enhanced production of H_2_O_2_ was detected, it did not lead to oxidative damage of membrane-forming phospholipids. However, CecA elevated MDA level at a concentration of 3.125 μg/mL in Poly I:C-induced inflammation, which might result from unknown oxidative events different from the formation of H_2_O_2_, as it was not detected to elevate. Interestingly, similar observations were made in the case of HDP LRR11, as the peptide enhanced the generation of ROS when applied simultaneously with a pro-inflammatory agent; however, did not display a prooxidant effect when administered alone (53). Although our findings are contrary to the previously proven antioxidant activity of other cecropins (14–17), it was also found that cecropin 3 (52) and cecropin-like peptide Hp (2–20) (51) contributed to oxidative stress which was initially exerted as a host defense response against pathogens, but ended up displaying a detrimental impact on host cells (51). In addition, when investigating the anti-tumor activity of CecA, it was observed that the peptide led to the accumulation of ROS at its higher concentration in a human promyelocytic leukemia cell line (24). Moreover, CecA was found to promote excessive formation of ROS in *Candida albicans* also, which was considered an essential part of the antifungal activity of the peptide (54, 55). Taking our results together, the effect of CecA on redox homeostasis is difficult to evaluate, and it might have required the investigation of further parameters, the lack of which is a limitation of our study. Therefore, further *in vitro* and *in vivo* studies are needed to examine the detailed effects of CecA on the cellular oxidative state, to which our study may provide useful information.

In summary, our study confirms that HDPs are versatile biological regulators of the immune response and inflammatory pathways, as they fight against pathogens in a complex manner, possibly contributing to their future application as immunomodulatory antimicrobial agents. However, to achieve this, HDPs need to be examined widely at the cellular level and in different species, especially concerning their use in livestock farming, for which fewer studies are available.

## 5 Conclusion

The present study aimed to investigate the effects of CecA on the inflammatory response and redox homeostasis of a primary chicken hepatocyte-non-parenchymal cell co-culture. According to our results, CecA seems to have no harmful effects on the viability of hepatic cells when applied at its lower concentrations; however, the use of its higher concentrations might result in cell membrane damage. It can also be stated that CecA possesses a multifaceted impact on the host cells’ immune response, as it was able to influence the levels of IL-6, IL-8, IFN-γ, IL-10, and TGF-ß1. Even though, based on our results, CecA cannot be considered purely anti-inflammatory, it is suggested to maintain the hepatic inflammatory homeostasis in Poly I:C-triggered immune response. In addition, the examination of the effects of CecA on the cellular redox state showed that the oxidative parameters were not affected in most cases of CecA exposure, even so, further studies are required to understand its action. To conclude, CecA offers more than a simple antibacterial effect, and it might be a promising candidate for the future design and development of antimicrobial agents, thereby contributing to the reduction of the use of conventional antibiotics and antibiotic resistance.

## Data availability statement

The datasets presented in this study can be found in online repositories. The names of the repository/repositories and accession number(s) can be found at: 10.6084/m9.figshare.23997939.

## Ethics statement

The animal study was approved by Local Animal Welfare Committee and Government Office (number of permission: GK-419/2020; date of approval: 11 May 2020). The study was conducted in accordance with the local legislation and institutional requirements.

## Author contributions

RM: Conceptualization, Data curation, Formal analysis, Investigation, Methodology, Software, Visualization, Writing – original draft, Writing – review & editing. CS: Investigation, Methodology, Writing – review & editing. MM: Formal analysis, Investigation, Methodology, Validation, Writing – review & editing. PT: Investigation, Methodology, Writing – review & editing. JV: Investigation, Methodology, Writing – review & editing. ÁK: Methodology, Software, Validation, Writing – review & editing. ZN: Investigation, Methodology, Writing – review & editing. GM: Conceptualization, Formal analysis, Investigation, Methodology, Validation, Writing – review & editing.
